# Early detection of ICU-acquired infections using high-frequency electronic health record data

**DOI:** 10.1186/s12911-025-03031-6

**Published:** 2025-07-21

**Authors:** Meri R. J. Varkila, Giacomo Lancia, Maarten van Smeden, Marc J. M. Bonten, Cristian Spitoni, Olaf L. Cremer

**Affiliations:** 1 Julius Center for Health Sciences and Primary Care, University Medical Center Utrecht, Utrecht University, Utrecht, Netherlands; 2 Department of Intensive Care Medicine, University Medical Center Utrecht, Utrecht, Netherlands; 3 Department of Mathematics, University Utrecht, Utrecht, Netherlands

**Keywords:** Critical illness, ICU, Infection, Nosocomial, Machine learning

## Abstract

**Background:**

Nosocomial infections are a major cause of morbidity and mortality in the ICU. Earlier identification of these complications may facilitate better clinical management and improve outcomes. We developed a dynamic prediction model that leveraged high-frequency longitudinal data to estimate infection risk 48 h ahead of clinically overt deterioration.

**Methods:**

We used electronic health record data from consecutive adults who had been treated for > 48 h in a mixed tertiary ICU in the Netherlands enrolled in the Molecular Diagnosis and Risk Stratification of Sepsis (MARS) cohort from 2011 to 2018. All infectious episodes were prospectively adjudicated. ICU-acquired infection (ICU-AI) risk was estimated using a Cox landmark model with high-resolution vital sign data processed via a convolutional neural network (CNN).

**Results:**

We studied 32,178 observation days in 4444 patients and observed 1197 infections, yielding an overall infection risk of 3.5% per ICU day. Discrimination of the composite model was moderate with c-index values varying between 0.64 (95%CI: 0.58–0.69) and 0.72 (95%CI: 0.66–0.78) across timepoints, with some overestimation of ICU-AI risk overall (mean calibration slope 0.58). Compared to 38 common features of infection, a CNN risk score derived from five vital sign signals consistently ranked as a strong predictor of ICU-AI across all time points but did not substantially change risk prediction of ICU-AI.

**Conclusion:**

A dynamic modelling approach that incorporates machine learning of high-frequency vital sign data shows promise as a continuous bedside index of infection risk. Further validation is needed to weigh added complexity and interpretability of the deep learning model against potential benefits for clinical decision support in the ICU.

**Supplementary Information:**

The online version contains supplementary material available at 10.1186/s12911-025-03031-6.

## Background

As nosocomial infections remain a major cause of morbidity and mortality in hospitals and intensive care units (ICUs) worldwide, methods for early detection of (severe) infections have been much pursued by clinical researchers and electronic health record (EHR) vendors alike. Numerous sepsis prediction algorithms have been proposed in the literature, including early warning scores, conventional regression models and novel machine learning models [1–5]. However, accurate detection of infection at a sufficiently early stage remains difficult.

Challenges in predicting infection relate to its insidious onset as well as its associated diagnostic uncertainty. Multiple infections can also exist concurrently or occur in close succession. Most published detection algorithms for use in critically ill patients, however, focus on predicting the presence of sepsis or septic shock upon ICU presentation and overlook consecutive infections. In addition, existing methods have mainly relied on static modelling and thus fail to fully optimize the wealth of longitudinal data generated in an ICU environment. Although this information can be exploited by dynamic modeling approaches, subtle trends contained in high-resolution EHR signals, such as vital sign data, are challenging to capture as informative inputs for prediction algorithms [6]. Furthermore, the degree to which various symptoms and signs contribute to the infection risk prediction can vary over time. Modern methods that can harness such temporal dynamics could improve timely detection of infections in the ICU and help physicians to better manage these complications.

In this study, we explore a dynamic modelling framework to continuously update predictions as time progresses and investigate if a deep learning algorithm trained to extract hidden temporal patterns from physiological signals can improve timely detection of ICU-acquired infections (ICU-AI).

## Materials and methods

### Study design

This study was nested within the framework of the Molecular Diagnosis and Risk Stratification of Sepsis project (ClinicalTrials.gov identifier NCT01905033, date January 2011), a prospective ICU cohort study, for which the institutional review board approved an opt-out method of informed consent (protocol number 10–056 C, date: June 16, 2010, study title: “Molecular Diagnosis and Risk Stratification of Sepsis (MARS)”) whereby participants and family members were notified of the study by a brochure with an attached opt-out card that was provided at ICU admission. The current analysis was additionally reviewed by the Medical Research Ethics Committee Utrecht and deemed exempt from the need for consent to participate in accordance with national regulations (protocol number 19–241 C, date April 3, 2019). All procedures were conducted in accordance with the Helsinki Declaration of 1975. For the current analysis, we obtained longitudinal EHR data from consecutive subjects > 18 years having an ICU stay > 48 h who were admitted to the medical-surgical tertiary ICU of one of the participating centers (University Medical Center Utrecht in the Netherlands) between January 2011 and December 2018. This study follows the Transparent Reporting of a multivariable prediction model for Individual Prognosis or Diagnosis using regression modelling or machine learning (TRIPOD + AI) reporting guidelines [7].

### Variables and outcomes

The main outcome of this study was onset of suspected ICU-AI. Infections were considered ICU-acquired if they had an onset that occurred > 48 h after ICU presentation. All suspected episodes had been previously recorded as part of the prospective MARS project [8]. Reference time of infection diagnosis was defined by the start of empirical antibiotic therapy, or the collection of a blood culture that (later) became positive and was followed by antibiotic therapy, whichever occurred first.

We selected a wide range of candidate predictor variables based on literature review, a priori clinical expertise, and observed prevalence in the study population. These variables included static admission characteristics reflecting baseline risk of infection, and dynamic features representing changes in a patient’s clinical condition over time (such as laboratory measurements, physiological response and organ function parameters; full list in Supplementary Material 2). In addition, high-resolution time series data from five vital sign signals (i.e., heart rate, mean arterial blood pressure, pulse pressure, respiratory rate, and arterial oxygen saturation) were extracted from the ICU patient data management system at 1-minute intervals.

### Model framework and data-preprocessing

**Fig. 1:**
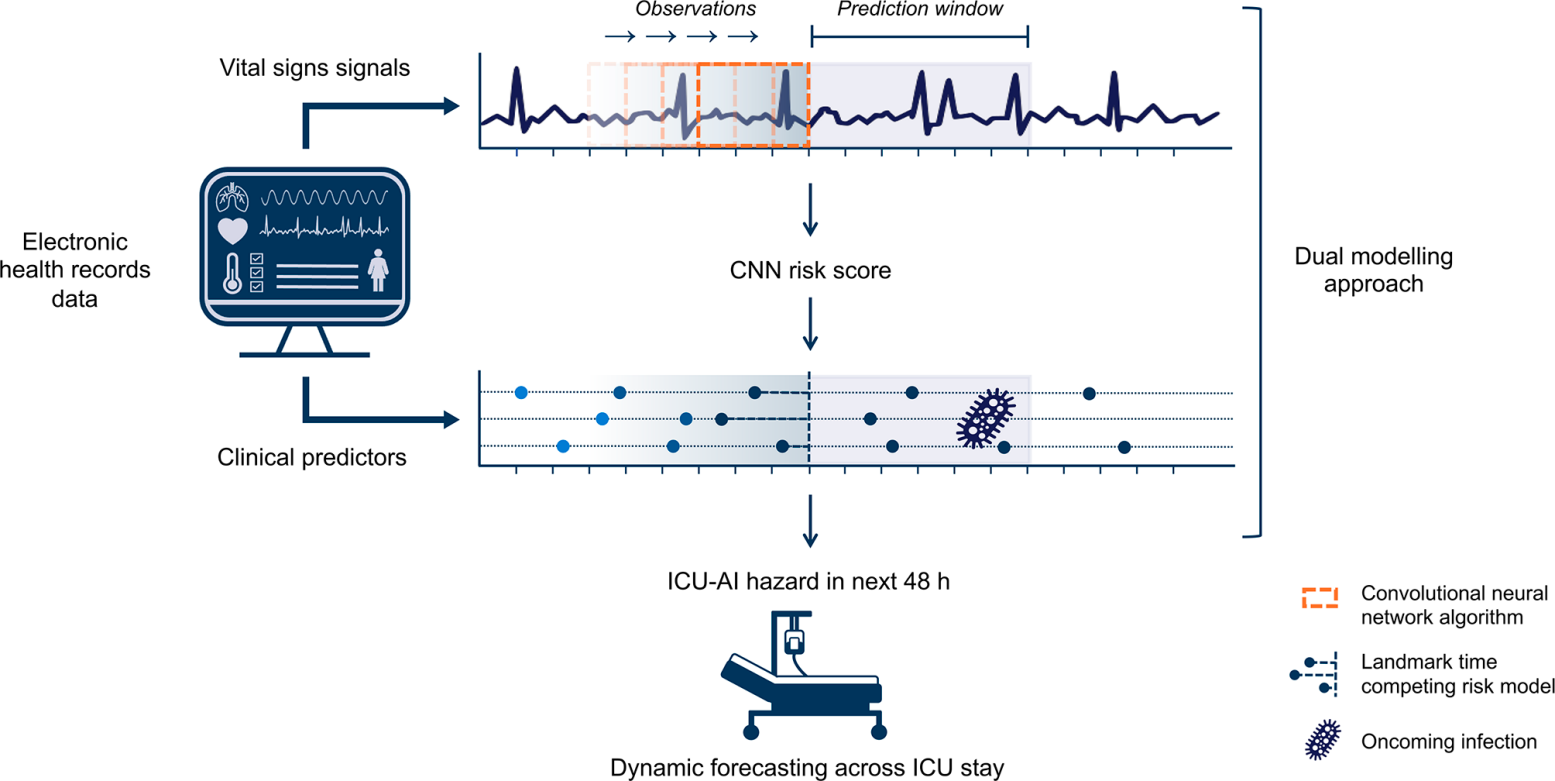
Modelling framework. Data were obtained from a large prospective ICU cohort. We derived a risk score using deep learning (CNN) to identify patterns in vital signs time-series data associated with impending infection. This CNN risk score was subsequently incorporated into a comprehensive landmarking competing risk Cox model that integrates both static and time-varying clinical predictors of infection as they become available during ICU stay. The model produces 48-hour infection risk estimates that update in real-time throughout a patient’s admission. All data generated during the first 24 h of ICU stay was excluded and observation time was initiated at 24 h after ICU presentation. Predictions began at the first landmarking timepoint, set at 48 h after ICU presentation. ICU-AI; intensive care unit-acquired infection. CNN; convolutional neural network

We used a landmarking framework to effectively model time-series data and obtain dynamic predictions throughout ICU admission. A landmark point refers to a moment in time *t*_LM_ at which risk predictions are made using the most recent information collected up until that timepoint. Time-series data were partitioned into equally spaced 8-hour intervals, whereas the prediction window was fixed at 48 h from *t*_LM_ (Fig. 1). We opted for 8-hour intervals to reflect a typical daily workflow in the ICU and provide ample time for clinically meaningful changes in patient status between consecutive predictions. The 48-hour prediction window was chosen as to generate clinically meaningful and actionable predictions. Furthermore, we excluded all data generated during the first 24 h and subsequently defined the first landmarking timepoint at 48 h after ICU presentation. Once an outcome event (i.e., onset of ICU-AI) had occurred, we excluded all data observed during the 48 h following this event. Thus, the sliding prediction window was “reset” and landmarking resumed at 8-hourly intervals as described above. Such reinitiated observation windows were treated as independent data during model development, with each time series representing a new period of event-free ICU stay.

Missing data were imputed using appropriate techniques. Further details about data pre-processing can be found in the electronic supplement.

### Model development

#### Landmarking survival model

We estimated the risk of ICU-AI onset within the next 48 h using a landmark time competing risk Cox (LMCR) model. We constructed a series of independent models for each landmark which optimally leveraged the predictive information contained in the patient subset at risk at that time point (i.e., patients still residing in the ICU and at risk of future infection at each 8-hourly landmark). Competing outcome events for these models were death and discharge from ICU. The survival probability of ICU-AI within a 48-hour time horizon was thus estimated conditional to surviving event-free until each landmark time. Subsequently, we fit the ensemble model using a stacked data set that was stratified on the landmark times [9, 10]. This allowed us to obtain hazard ratios (HR) for the 48-hour prediction window across all landmarking points. By observing changes in HR over time, we could make inferences about the association between predictors and the evolving risk of ICU-AI.

The final model included linear and quadratic terms for landmarking time to account for changing infection risk with increasing duration of ICU stay. Since commonly used methods for covariate selection (e.g., backward elimination, forward selection, or regularization and shrinkage methods) are inappropriate for dynamic landmark models (as different predictors may be selected at different time points) and do not guarantee optimal model performance overall [11], we elected not to perform any predictor reduction.

#### Deep learning algorithm

To assess whether temporal patterns in physiological signals can improve detection of ICU-AI we trained a deep learning algorithm using high-resolution vital sign data. Deep learning is a subset of machine learning that utilizes multilayer artificial neural networks to extract representations from raw data. For the present study, a convolutional neural network (CNN) was trained using high-resolution time series data of five selected vital signs as one-dimensional inputs. A CNN propagates these inputs through several hidden layers (i.e., convolutional layer, pooling layer, and dropout layer) and generates a feature map of areas where patterns are detected. The feature map is then flattened into an array and propagated via a fully-connected output layer with a sigmoid activation function. This returns a positive number between 0 and 1 that can be interpreted as the probability of infection onset within the next 48 h. The optimal configuration for this deep learning algorithm was selected by evaluating the mean AUC values in 5-fold cross validations using various combinations of hyperparameters. This yielded a network consisting of a sequence of five sets of 3 hidden layers. Additional mathematical details about the LMCR model and the architecture of the final CNN have been presented in more detail previously [12].

### Model evaluation

We used a cumulative incidence function (with death and discharge as competing risks) to estimate the absolute risk of ICU-AI occurrence within the 48-h prediction window [10]. Model performance across different landmark times was evaluated using repeated 5-fold cross-validations. To assess the added value of the CNN approach, we employed a method where the infection probability (i.e., the final CNN output) was used as an ancillary predictor in the landmark model. Performance of the LMCR-base model was subsequently compared to a LMCR model which also included this CNN-risk score (from here on referred to as the Deep-LMCR model). We evaluated discriminatory ability using Harrell’s c-index after administrative censoring of competing events [13, 14], with higher indexes signaling better performance. Classification accuracy was evaluated using the Brier score (which is similar to mean squared error), with lower scores indicating better performance [15]. For all aforementioned estimates, we report the mean over five folds along with 95% confidence intervals (95%CI). Calibration in the presence of competing risks was evaluated using calibration curves that compared observed events with expected events based on cumulative incidence estimates [16]. Subsequently, calibration slopes and intercepts were calculated [17]. The likelihood-ratio test was used to compare goodness of fit between the LMCR-base and Deep-LMCR models. Finally, to assess feature importance of predictors over time, we used heatmaps visualizing the ranking of individual variables according to their Wald Χ^2^ statistics and their relative contributions to the c-index.

In sensitivity analyses, we restricted the prediction window from 48 to 24 h. We also trained a two-dimensional CNN model to harvest potentially complementary features from the time series signals that could not be modeled by the original one-dimensional network. All analyses were performed using R Studio version 1.3 (R Core Team 2013, Vienna, Austria), and Python software (Python Software Foundation, Python Language Reference, version 2.7).

## Results

**Fig. 2:**
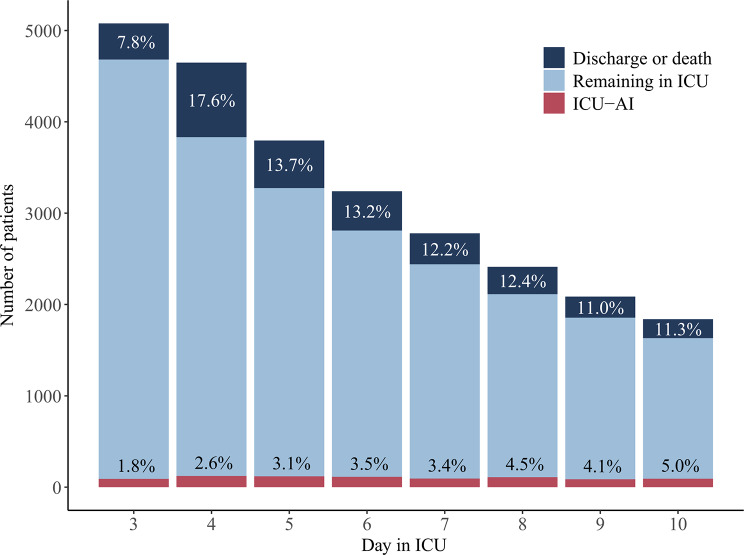
Study outcomes across time in ICU. Columns show total number of patients (i.e. the study population size) by day in ICU, with the bars split according to the proportion of patients (%) that experienced the main study outcome (ICU-AI) or a competing outcome event on a given ICU day

We studied 5075 ICU admissions in 4444 unique individuals, amounting to 32,178 observation days available for model development (Supplementary Material 2). Case fatality was 417 (8.2%) by day 10. We observed 1197 episodes of ICU-AI during 954 (18.8%) admissions. 194 (4.4%) patients experienced recurring infection events. The first episode of ICU-AI occurred after a median of 7 (interquartile range (IQR) 5–9) days, whereas recurring infection occurred after a median of 14.5 (IQR 12–18) days. Risk of infection onset remained relatively constant over time (daily incidence rate 3.5%, 95%CI 2.8 to 4.2%; Fig. 2).

Patients who developed ICU-AI were older, more frequently immunocompromised, more often had a surgical reason for admission, and were more severely ill at the time of ICU admission compared to their counterparts without (ever) infection (Table 1). The median length of ICU stay was also significantly longer (median 14 [IQR 9–23] vs. 5 [IQR, 3–8] days; *p* < 0.001) and ICU mortality higher (28.0% vs. 12.7%; *p* < 0.001).

**Table 1:** Patient characteristics & outcomes in patients with ICU-acquired infection or not

	No infection *N* = 4121		ICU-AI *N* = 954	
**Patient characteristics**				
Age, median (IQR)	62	(50–71)	61	(52–70)
Male, N (%)	2567	(62.3)	681	(71.4)
Chronic comorbidities				
Diabetes Mellitus, N (%)	683	(16.6)	134	(14.0)
Immunodeficiency, N (%)	650	(15.8)	180	(18.9)
Malignancy, N (%)	698	(16.9)	139	(14.6)
Cardiovascular insufficiency, N (%)	738	(17.9)	176	(18.4)
Renal insufficiency, N (%)	338	(8.2)	93	(9.7)
Respiratory insufficiency, N (%)	317	(7.7)	77	(8.1)
Surgical admission, N (%)	1736	(42.1)	460	(48.2)
Previously admitted to ICU, N (%)	649	(15.7)	125	(13.1)
Sepsis at ICU admission, N (%)	1833	(44.5)	347	(36.4)
APACHE-IV score, median (IQR)	77	(60–98)	83	(65–104)
SOFA-score, median (IQR)	7	(4–9)	8	(6–10)
***ICU outcomes***				
Total ICU-AI, N			1197	
Day of first ICU-AI, median (IQR)			7	(5–11)
Site of infection, N (%)				
Abdominal			102	(8.5)
Bacteremia*			40	(3.3)
Catheter-related bloodstream infection *			272	(22.7)
Other cardiovascular			31	(2.6)
Neurological			72	(6.0)
Pulmonary			540	(45.1)
Other			140	(11.7)
ICU length of stay, median days (IQR)	5	(3–8)	14	(9–23)
ICU mortality, N (%)	523	(12.7)	267	(28.0)

* Bacteremia events were limited to primary bloodstream infections without an identifiable source during adjudication. Secondary bacteremia events, including catheter-related bloodstream infections, are listed under their respective infection sites

Abbreviations: APACHE Acute Physiology and Chronic Health Evaluation; ICU-AI Intensive Care Unit Acquired Infection; IQR Interquartile range; N Number; SOFA Sequential Organ Failure Assessment

**Fig. 3:**
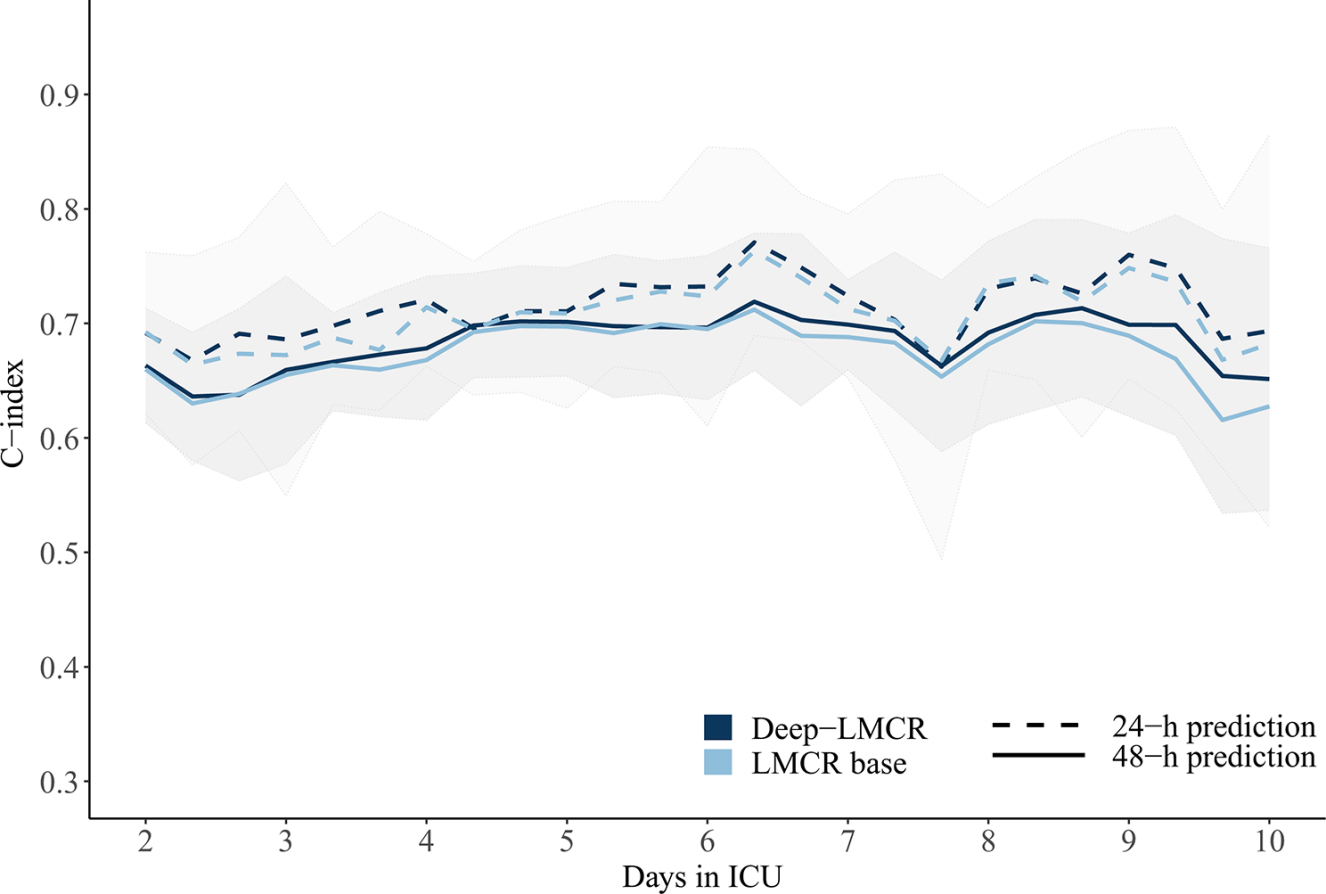
Cross-validated model performance across time in ICU. C-index values are depicted across landmarking time points. Solid lines show the c-index values of models trained to predict ICU-AI with a 48-hour prediction window. Dashed lines show c-index values of the models trained with a 24-hour prediction window. The light blue line shows c-index values of the landmarking base model and dark blue line indicates c-index of the deep-LMCR model. Shaded bands depict 95% confidence intervals c-index values for the 24-h and 48-h deep-LMCR models. ICU-AI; intensive care unit-acquired infection. LMCR; landmark time competing risk Cox model

The LMCR-base model was fitted using 9 time-invariant and 29 time-varying covariates. A list of measurements with frequency of missing data is shown in the Supporting information (Supplementary Material 2). The LMCR-base model had mediocre discriminatory ability overall (C-index 0.69, 95% confidence interval (CI): 0.68–0.69). This estimate did not change with cross-validation (C-index 0.69, 95%CI: 0.65–0.72). When evaluated across the various landmark points (i.e., between ICU days 2 and 10), concordance varied between 0.62 (95%CI: 0.49, 0.73) and 0.71 (95%CI: 0.64, 0.78) (Fig. 3). Prediction accuracy for the LMCR-base model was good (Brier score 0.05; 95%CI: 0.05–0.06). Calibration assessment across various prediction moments indicated some overestimation of the probability of ICU-AI overall (calibration intercepts between − 0.02 and − 0.44), most notably in patients with highest predicted risk (calibration slopes between 0.4 and 0.66) (Supplementary Material 2).

**Fig. 4:**
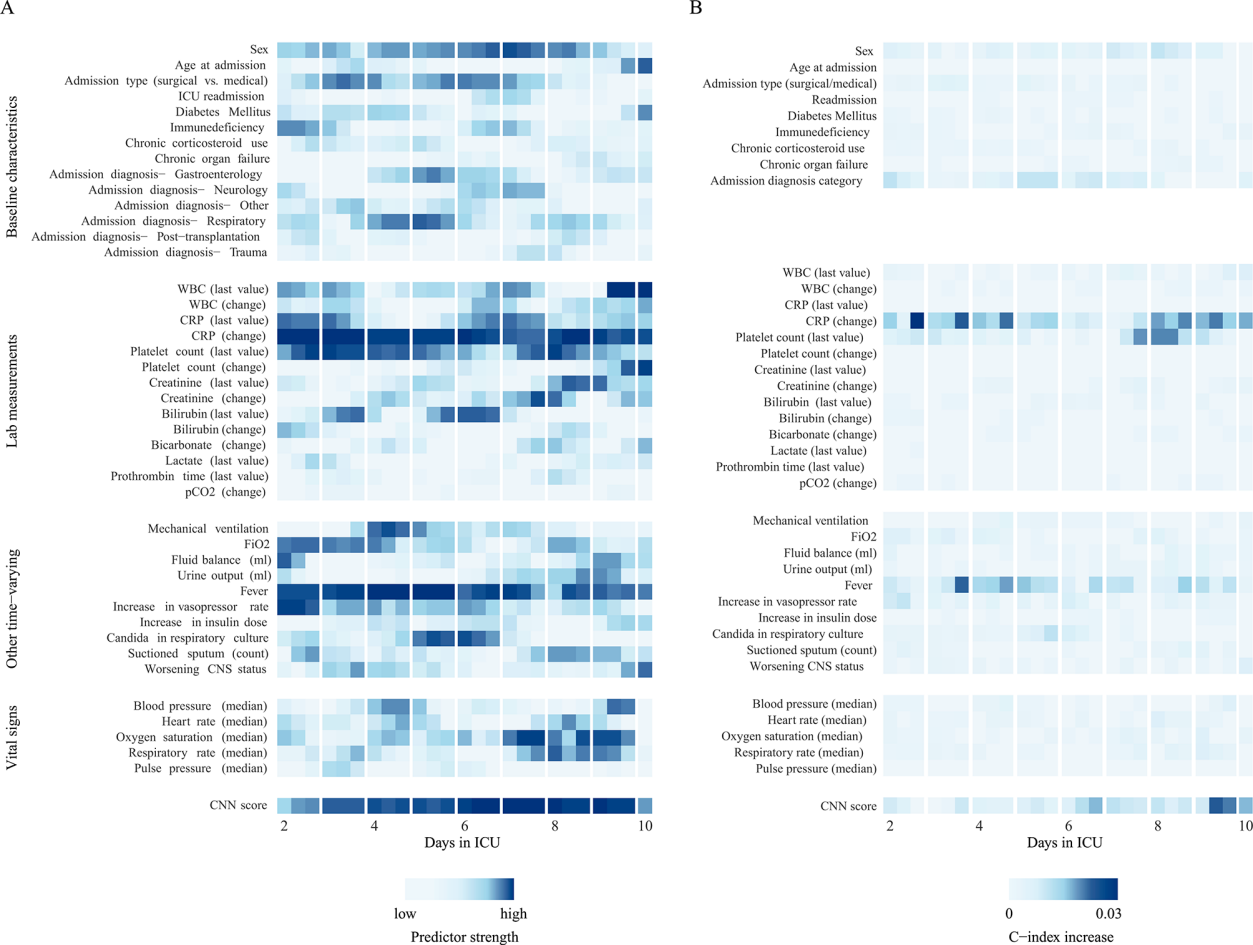
Strength and impact of input features for the prediction of 48-hour infection risk across days in ICU. The left column depicts the relative strength of all 39 predictors on infection risk across landmarking time points ranked according to the Wald X^2^ statistic. The right column shows the relative contribution of individual predictors to the c-index across landmarking times. Darker shades of blue indicate higher predictor strength or larger impact on the c-index. CNN; convolutional neural network. CNS; central nervous system. CRP; c-reactive protein. FiO2; fraction of inspired oxygen. ICU; intensive care unit. pCO2; partial pressure of carbon dioxide. WBC; white blood cell count

Regression coefficients for all predictors used in the LMCR-base model are provided in Supplementary Material 2. Predictors that consistently displayed high predictive strength included a rise in CRP, fever, and lower platelet counts. However, the strength of many predictors appeared to vary across time points (Fig. 4). For example, the association of lower oxygen saturation and higher respiratory rate with ICU-AI risk increased after 7 days of ICU stay.

When integrated into the LMCR-base model, the CNN risk score emerged as one of the strongest predictors of ICU-AI overall (cause-specific HR 8.62, 95%CI: 5.22–14.23, beta-coefficient 2.2, *p* < 0.001), which retained its impact across various time points in the ICU (Fig. 4, Supplementary Material 2). Furthermore, according to the likelihood-ratio test, adding the CNN risk score to the LMCR-base model could improve prediction of ICU-AI (*p* < 0.001). However, despite these findings, incorporating the CNN risk score into the model did not significantly improve overall predictive performance in practice (c-index 0.69; 95%CI: 0.68–0.69 versus 0.69, 95%CI: 0.68–0.70 for the LMCR-base and Deep-LMCR models, respectively). Across prediction moments, discriminative ability showed the greatest improvement by the addition of the CNN risk score at later landmarking times with c-indexes for the Deep-LMCR varying between 0.64 (95%CI: 0.58–0.69) and 0.72 (95%CI: 0.66–0.78) (Fig. 3). The Brier score for the Deep-LMCR model was equivalent to that of the base model. Similarly, overall calibration was unchanged (Supplementary Material 2).

A sensitivity analysis, in which the prediction window for infection was reduced to 24 h, showed better discrimination compared to models using a 48-hour prediction window (global c-index 0.71 (95%CI: 0.70, 0.72), and 0.72 (95%CI: 0.71, 0.73) for the 24-hour LMCR-base and Deep-LMCR models, respectively) (Fig. 3). However, addition of a two-dimensional CNN score to the landmarking model did not have additional predictive value (data not shown).

## Discussion

Early, potentially even presymptomatic identification of critically ill patients who are developing ICU-AI is essential for the timely initiation of diagnostic and therapeutic procedures. Leveraging expansive data from a prospective cohort of critically ill patients, we developed a dynamic prediction model that integrates new clinical information from the EHR as it becomes available in real time and is adaptive to changes in a patient’s clinical condition during (at least) the first 10 days of ICU stay. In this study, while clinical predictors varied considerably over time, the CNN risk score consistently emerged as a strong predictor of ICU-AI and outperformed most other inputs from EHR. The addition of the CNN risk score, however, did not markedly influence the discriminative ability of our model.

Our findings are in accordance with previous studies that have demonstrated that machine learning methods can achieve good accuracy in sepsis detection, albeit at prediction windows of hours rather than days [1, 2, 18, 19]. We hypothesized that predictor values close to the clinical event may be influenced by reverse causation, as well as a clinicians’ actions if signs of deterioration were already recognized and would thus contribute to less meaningful predictions. We chose to estimate the risk of oncoming ICU-AI using a clinically relevant and actionable prediction window of 48 h. Despite these demands, our model demonstrated reasonable discrimination based on an overall *dynamic* c-index 0.69 (95%CI 0.68–0.70). In interpreting the c-index, it should be noted that this measure reflects ability of the model to assign higher predictions to patients about to experience ICU-AI in the next 48 h compared to patients who do not and that the sets of “cases” and “controls” change over time (i.e. a control can becomes a case at another prediction moment if ICU-AI occurs later). As such, discrimination should not be directly compared to reported AUCs of traditional static models or real-time prediction models that report *encounter-* or *patient-level* AUCs. Our sensitivity analysis demonstrated that reducing the predictive time horizon from 48 to 24 h improved discrimination by as much as 6%. Indeed, in a recent meta-analysis of 111 machine learning models expected AUROC values < 0.6 were consistently observed for various modeling methodologies as the prediction window increased beyond 10 h before sepsis onset [2]. Our results obtained at a 48-hour prediction window thus compare favorably to these results, although external validation is still necessary.

Paradoxically, despite its strong association with ICU-AI, the CNN risk score demonstrated limited incremental value for improving prediction. The c-index is a widely used metric to gage a model’s ability to separate cases from controls, however, it has limited ability to reflect change if the improvement concerns a specific sub-group of patients [20, 21]. Whether a CNN risk score could identify high-risk individuals among specific patient populations warrants investigation in future studies. It is also possible, however, that the CNN risk score reflects information that is already encompassed by other predictors in the model. Deciphering which inputs and patterns contribute to predictions by a model often likened to a black box presents a considerable challenge. The added complexity and limited interpretability of a deep learning algorithm and computational resources required to develop and implement such a tool should therefore be carefully weighed against any gains in data extraction. As computationally simple approaches that readily quantify relationships within data, dynamic statistical models, such as landmarking survival analysis, joint models or Markov-chain multistate transition models, present attractive alternatives for the early detection of infection in the ICU [22, 23].

Ultimately, the clinical relevance and utility of a model depend on the context and practical implications of calculated predictions [24]. The observed 48-hour mean risk of new onset ICU-AI in our cohort at any given time was 7.7%, while the total cumulative risk of developing ICU-AI across ICU stay was 18.8%. Performing diagnostic bundles or molecular tests when model predictions indicate increased risk of ICU-AI could provide additional evidence of infection and assist in further decision making [25–28]. With the Deep-LMCR model, 20% of predictions generated an estimated 48-hour risk of ICU-AI of higher than 10%. In patients with oncoming ICU-AI the first alert using this (arbitrary) threshold of 10% came median 64 (IQR 24, 112) hours before onset of infection. If evaluating patients at this threshold would lead to interventions that prevented or improved infectious outcomes in 70% of cases, the cumulative incidence of ICU-AI could be reduced from 18.8 to 9.4%. At the same time, however, 30% of ICU patients would be evaluated needlessly.

We acknowledge some limitations of our study. Our models were developed using data from a single center and were not validated using an external cohort. Instead, we chose to perform cross-validation to account for overoptimism of our estimates. Our ICU also used selective digestive tract decontamination throughout the study period, which may impact incidence of ICU-AI and not reflect the infectious burden in other ICU settings. Although the aim of our study was not to the investigate the potential utility of a prediction algorithm in clinical practice, in which case external validation is essential, lack of external validation and the study setting may affect generalizability of our results.

## Conclusion

In this study, we applied a landmarking approach to predict onset of ICU-AI up to 48 hours in advance of clinical deterioration using predictors that can readily be extracted from an EHR in conjunction with a neural network algorithm trained on high-resolution time series data. Even though the deep learning risk score emerged as one of the strongest and most consistent predictors among 39 features contained in our landmark model, the deep learning approach was not able to capture meaningful ‘hidden’ information from the vital sign streams that was not already contained in the comprehensive set of early signs and symptoms of infection. This illustrates the need to always validate novel machine learning approaches against classical predictions based on readily available clinical information.

## Electronic supplementary material

Below is the link to the electronic supplementary material.

Supplementary Material 1

Supplementary Material 2

Supplementary Material 3

## Data Availability

Python codes and modules used for CNN development and validation are available via a GitHub repository at https://github.com/glancia93/ICUAI-dynamic-prediction. Data privacy policies prohibit deposition of individual level data to public repositories and the public sharing of data for unknown purposes. Upon contact with the authors an institutional data transfer agreement may be established, and data shared if the aims of data use are covered by ethical approval.

## Declarations

### Peer review

The peer review reports can be found at 10.1186/s12911-025-03031-6.

## Abbreviations

ICU: Intensive care unit
EHR: Electronic health record
ICU-AI: Intensive care unit acquired infection
MARS: Molecular Diagnosis and Risk Stratification of Sepsis
LMCR: Landmark time competing risk
CNN: Convolutional neural network
IQR: Interquartile range
CI: Confidence interval
