# Supplementary material for: Early detection of ICU-acquired infections using high-frequency electronic health record data
Source: BMC Med Inform Decis Mak. 2025 Jul 21;25:273. doi: 10.1186/s12911-025-03031-6 (PMC12278606; doi:10.1186/s12911-025-03031-6)
Supplement: Supplementary file 1 — Supplementary Material 1 [file 12911_2025_3031_MOESM1_ESM.zip › 96dbfd30-ff69-4a88-93a4-4f1f84e6098a - report round 1.docx]

**Reviewer 1**

Submitted: **29 Jun 2024**

Feedback for the author(s)

This is a prediction model with high clinical relevance and the study developed A dynamic modelling approach that incorporates machine learning of high-frequency vital sign data shows promise as a continuous bedside index of infection risk. Further validation is needed to weigh added complexity and interpretability of the deep learning model against potential benefits for clinical decision support in the ICU. I have a few comments as follows:

1. there is a lack of population selection section; how did you construct the cohort? In the present case, those with infection on admission should be excluded.

2. will you consider TRIPOD-AI checklist?

3. There are many factors related to the outcome being identified in the model, however their causal relationship is largely unknown; I would suggest discussion on this point in the context of causal inference (https://doi.org/10.1016/j.lers.2022.10.002) since the causal association can have significant impact on clinical management.

4. do you have data on the site of infection? In table 1, bacteremia and CRBSI is not mutually exclusive.

5. In competing risk analysis, the ICU discharge can be a competing risk for the infection.

6. The most important risk factor for ICU-AI is the long stay in ICU; with each day increase in ICU, the cumulative risk can increase. This should be accounted for during modeling.

**Reviewer 2**

Submitted: **06 Mar 2025**

Feedback for the author(s)

PEER REVIEWER ASSESSMENTS:

OBJECTIVE - Full research articles: is there a clear objective that addresses a testable research question(s) (brief or other article types: is there a clear objective)?

Yes - there is a clear objective

DESIGN - Is the current approach (including controls and analysis protocols) appropriate for the objective?

No - there are minor issues

EXECUTION - Are the experiments and analyses performed with technical rigor to allow confidence in the results?

No - there are minor issues

STATISTICS - Is the use of statistics in the manuscript appropriate?

Yes - appropriate statistical analyses have been used in the study

INTERPRETATION - Is the current interpretation/discussion of the results reasonable and not overstated?

Yes - the author's interpretation is reasonable

OVERALL MANUSCRIPT POTENTIAL - Is the current version of this work technically sound? If not, can revisions be made to make the work technically sound?

Probably - with minor revisions

PEER REVIEWER COMMENTS: GENERAL COMMENTS: The authors present a dynamic prediction model that leveraged high-frequency longitudinal data to estimate ICU acquired infection risk 48 hours ahead of clinical deterioration. Machine learning models are increasingly becoming highly relevant in predicting risk for severe disease and also in diagnosis . The study uses robust methodology to estimate dynamic risk of infections. The data have high clinical relevance and highlights the need for updating AI models with clinical data.

REQUESTED REVISIONS: How was ICU AI infection defined or identified ? Line 158: Landmarking survival model estimated the risk of ICU-AI onset within the next 48 hours, was it next 48 hours after 48 hours of ICU presentation or from ICU presentation ? Line 161: “the patient subset at risk at that time point” does this mean at every 8 hour time period when the patient in in risk of future infection . How was case fatality and ICU mortality rates calculated? What was the median time for onset of recurring infections? Line 239: who were the high-risk patients ? Predictors that showed higher strength when were they measured ?
