# Supplementary material for: Early detection of ICU-acquired infections using high-frequency electronic health record data
Source: BMC Med Inform Decis Mak. 2025 Jul 21;25:273. doi: 10.1186/s12911-025-03031-6 (PMC12278606; doi:10.1186/s12911-025-03031-6)
Supplement: Supplementary file 1 — Supplementary Material 1 [file 12911_2025_3031_MOESM1_ESM.zip › 96dbfd30-ff69-4a88-93a4-4f1f84e6098a - report round 2.docx]

**Reviewer 1**

Submitted: **17 Mar 2025**

Feedback for the author(s)

my previous comments are well addressed.

**Reviewer 2**

Submitted: **24 Mar 2025**

Feedback for the author(s)

PEER REVIEWER ASSESSMENTS: OBJECTIVE - Full research articles: is there a clear objective that addresses one or several testable research questions? (Brief or other article types: is there a clear objective?)

Yes - there is a clear objective

DESIGN - Is the current approach (including controls and analysis protocols) appropriate for the objective?

No - there are minor issues

EXECUTION - Are the experiments and analyses performed with sufficient technical rigor to allow confidence in the results?

No - there are minor issues

STATISTICS - Is the use of statistics in the manuscript appropriate?

Yes - appropriate statistical analyses have been used in the study

INTERPRETATION - Is the current interpretation/discussion of the results reasonable and not overstated?

Yes - the author's interpretation is reasonable

OVERALL MANUSCRIPT POTENTIAL - Has the author addressed your concerns sufficiently for you to now recommend the work as a technically sound contribution? If not, can further revisions be made to make the work technically sound?

Probably - with minor revisions

PEER REVIEWER COMMENTS: GENERAL COMMENTS: The authors ahve tried to address most of my comments apart from comment 4 and 7, please see specific comments below

REQUESTED REVISIONS: The authors ahve not given a proper clarification about calculating mortality rate . While they can calculate CFR , mortality rate cannot be calculated from this type of study and hence needs to be removed . Comment 7 : authors states " The predictor “fever” was based on body temperature, which was defined as the presence of fever (>38 degrees Celsius) in the preceding 8 hours" preceding to what , was this a dynamic process or just measured once referring to preceding 8 hours to admission, this needs clarification ?
