# Supplementary material for: Early detection of ICU-acquired infections using high-frequency electronic health record data
Source: BMC Med Inform Decis Mak. 2025 Jul 21;25:273. doi: 10.1186/s12911-025-03031-6 (PMC12278606; doi:10.1186/s12911-025-03031-6)
Supplement: Supplementary file 2 — Supplementary Material 2 [file 12911_2025_3031_MOESM2_ESM.docx]

ADDITIONAL CONTENT

**Early detection of ICU-acquired infections using high-frequency electronic health record data**

Meri RJ Varkila, Giacomo Lancia, Maarten van Smeden, Marc JM Bonten, Cristian Spitoni, Olaf L Cremer

CONTENTS

Materials and Methods

APPENDIX A- Data Preprocessing of time series

APPENDIX B- Additional table 1 Predictor definitions

Results

APPENDIX C- Additional table 2 Missingness of values across ICU admissions before data preprocessing

APPENDIX D- Additional table 3 Model coefficients for Deep- LMCR model

APPENDIX E- Additional fig. 1 Flowchart of inclusions

APPENDIX F- Additional fig. 2 Calibration of LMCR base model

APPENDIX G- Additional fig. 3 Calibration of Deep- LMCR model

APPENDIX H- Additional fig 4 Ranking of individual predictors across landmark timepoints

APPENDIX A

**Data pre-processing of time series**

All signals were checked for outliers and errors using histograms and descriptive statistics. Values were bounded to clinically plausible values and artefacts were corrected when possible.

Data for landmarking model

All time-varying predictors were first organized into 8-hour non-overlapping time series bins to accommodate for different sampling frequencies of available data. Continuous variables with sampling frequencies higher than once every hour were first sampled into 1-hour time bins, by taking the mean values if multiple measurements were available. Subsequently, predictors were re-sampled into 8-hour bins by taking the median of the 1-hour bins. These included the following variables: mean blood pressure, heart rate, fever, SaO2, respiratory rate and FiO2 level.

Variables potentially measured less frequently than every 8 hours, such as laboratory measurements and microbiological culture results, were up-sampled to produce values for all landmarking points. Missing values between measurements, including absent datapoints due to up-sampling, were imputed by last observation carried forward (LOCF). Missing values occurring before the first actual measurement were imputed using multiple imputation. In addition to the last observation values, we derived aggregate predictors based on changes between landmarking times that could modify a patient’s perceived risk of infection. These included the unit change of laboratory measurements, increase in vasopressor rate, increase in insulin rate, fluid balance, total urine production, new onset delirium or worsening central nervous system scores, and number of times sputum was suctioned in within the last 8 hours. Time-fixed baseline variables were repeated at each landmarking timepoint. Landmark datasets were derived by intervals of 8 hours. We then selected the subset of patients still at risk at time *s* and censored administratively at *s*+*w.*

Data for deep learning models

Deep learning algorithms were trained using high resolution vital signs time series. These signals included heart rate, respiratory rate, mean arterial blood pressure, pulse pressure and oxygen saturation. Vital signs signal inputs considered, but not included in the deep learning algorithm due to large number of missing values were minute volume, end-tidal CO_2,_ and body temperature. Missing values in the selected vital signs time series were imputed using a zero-order spline. To further optimize the data for the deep learning algorithms, each individual patient’s ICU timeline was divided into a sequence of rolling 24-hour observation windows followed by a 48-hour prediction window. These windows were partially overlapping and shifted across time by 8 hours. For training of the algorithm, all observations windows that were followed by a prediction window where suspected infection did not occur were labelled as non-infected. If, however, a suspected infection occurred within the 48-hour prediction window, the observation window was labelled as infected. To avoid misclassification due to information leaking from infected windows into windows directly following infection, we excluded 48 hours of data immediately following each timestamp of infection. After this 48-hour recovery period, observation windows were further labelled as described above. Data for internal validation was created by splitting ICU admissions into a training dataset (80%) and a test dataset (20%) using the holdout method on the patient level (ie. data from each patients was only made available in either the test set or the training set).

APPENDIX B

**Additional table 1. Definitions of predictors included in LMCR-base model**

| **Predictor name** | **Predictor description** |
| --- | --- |
| **TIME FIXED** |  |
| Sex | Sex (male/female) |
| Age | Age at ICU admission |
| Admission type | Medical vs. surgical admission |
| Readmission | Previous ICU admission during current hospitalization period |
| APACHE admission diagnosis category | APACHE diagnostic category of ICU admission (cardiovascular/ gastrointestinal/ neurological / respiratory/post-transplantation/trauma/other) |
| Diabetes Mellitus | Medical history of diabetes mellitus |
| Immunedeficiency | Immunocompromised status defined as having acquired immune deficiency syndrome, the use of corticosteroids in high doses (equivalent to prednisolone of >75 mg/day for at least 1 week), current use of immunosuppressive drugs, current use of antineoplastic drugs, recent hematologic malignancy, or documented humoral or cellular deficiency |
| Chronic corticosteroid use | Chronic medication use: systemic corticosteroids |
| Chronic organ failure | Presence of chronic organ insufficiency with one of the following conditions documented in medical history:   - Chronic heart failure defined as medical history of chronic NYHA class 2-4 or documented ejection fraction <45% (on echography in 2 years prior to ICU admission) or orthopnea with chronic diuretic use - Severe cardiovascular insufficiency defined as angina or dyspnea in rest or during minimal exercise (NYHA IV) - Chronic renal insufficiency defined as chronically elevated serum creatinine >177 μmol/L or chronic dialysis - Chronic restrictive, obstructive or vascular pulmonary disease leading to severe functional impairment - Chronic liver failure with portal hypertension (with positive liver biopsy) and/or upper gastrointestinal bleeding due to portal hypertension and/or episode of hepatic encephalopathy/coma due to medical history of liver failure |
| **TIME VARYING** |  |
| Heart rate | Median of 1-hour mean heart rate (bpm) |
| Blood pressure | Median of 1-hour mean blood pressure, either invasive mean arterial blood pressure measurement or non-invasive cuff (mmHg) |
| Oxygen saturation | Median of 1-hour mean oxygen saturation (%) |
| Respiratory rate | Median of 1-hour mean respiratory rate (rpm) |
| Pulse pressure | Median of 1-hour mean pulse pressure (difference between systolic and diastolic blood pressure, mmHg) |
| Invasive mechanical ventilation | Last observed mechanical ventilation status (MV/no MV) |
| FiO2 | Last observed FiO2 (=inspired oxygen concentration) value in 8 hours preceding landmark time |
| Fever | Presence of fever in last 8 hours preceding landmark time (i.e. body temperature >38 degrees Celsius) |
| Fluid balance | Fluid balance (mL) over past 8-hours preceding landmark time |
| Urine output | Total urine output (mL) in 8 hour window preceding landmark time |
| Suctioned sputum | Total number of times sputum was suctioned and observed within 8 hour time window preceding landmark time |
| Worsening CNS status | Either decrease in consciousness (either decrease in GSC M-score or worsening RASS score) or onset of new delirium episode in past 8 hours |
| CRP (last value) | Last observed CRP (mg/L) |
| CRP (change) | Unit change in CRP relative to CRP 24 hours earlier (mg/L) |
| White blood cell count (last value) | Last observed white blood cell count (x10^9/L) |
| White blood cell count (change) | Unit change in white blood cell (WBC) count relative to WBC hours earlier (x10^9/L) |
| Platelet count (last value) | Last observed platelet count (x10^9/L) |
| Platelet count (change) | Unit change in platelet count relative to platelet count 24 hours earlier (x10^9/L) |
| Prothrombin time (last value) | Last observed prothrombin time (seconds) |
| Creatinine (last value) | Last observed creatinine (µmol/L) |
| Creatinine (change) | Unit change in creatinine relative to creatinine 24 hours earlier (µmol/L) |
| Total bilirubin (last value) | Last observed total bilirubin (µmol/L) |
| Total bilirubin (change) | Unit change in total bilirubin relative to bilirubin 24 hours earlier (µmol/L) |
| Bicarbonate (change) | Unit change of bicarbonate relative to bicarbonate 24 hours earlier (mmol/L) |
| pCO2 (change) | Unit change of pCO2 relative to pCO2 24 hours earlier (mmHg) |
| Lactate (last value) | Last observed lactate (mmol/L) |
| Increase in vasopressor rate | Increase in mean norepinephrine dose relative to previous 8-h window |
| Increase in insulin dose | Increase in mean insulin dose relative to previous 8-h window |
| Candida in respiratory culture | *Candida* species cultured in airway (result of most recent culture) |

Predictors considered, but not included in the final model were: BMI, admission source (community vs. hospital), recent hematologic malignancy, chronic statin use, new onset atrial fibrillation , fluid intake (ml), Gram+ in respiratory culture (result of most recent culture), Gram- in respiratory culture (result of most recent culture), Gram- in rectal culture (result of most recent culture), continued furosemide infusion, change in Reintam’s Gastrointestinal Score.

APPENDIX C

**Additional table 2. Missingness of values across ICU admissions (N=5075) before data preprocessing**

| **Input data** | **Sampling rate** | **Some values missing^a^,**  **N (%)** | **Never measured^b^,**  **N (%)** |
| --- | --- | --- | --- |
| Sex | Once | 0 (0) | 0 (0) |
| Age | Once | 0 (0) | 0 (0) |
| Surgical admission | Once | 0 (0) | 0 (0) |
| Readmission | Once | 0 (0) | 0 (0) |
| Diabetes Mellitus | Once | 0 (0) | 0 (0) |
| Immunedeficiency | Once | 0 (0) | 0 (0) |
| Chronic corticosteroid use | Once | 0 (0) | 0 (0) |
| Chronic organ failure | Once | 0 (0) | 0 (0) |
| APACHE admission diagnosis category | Once | 0 (0) | 0 (0) |
| CRP | 1/24 h | 1712 (33.7) | 29 (0.6) |
| White blood cell count | 1/24 h | 1724 (34.0 | 30 (0.6) |
| Platelet count | 1/24 h | 1710 (33.7) | 33 (0.7) |
| Prothrombin time | 1/24 h | 2948 (58.1) | 648 (12.8) |
| Creatinine | 1/24 h | 1579 (31.1) | 26 (0.5) |
| Total bilirubin | 1/24 h | 1778 (35.0) | 34 (0.7) |
| Bicarbonate | 1/24 h | 1626 (32.0) | 25 (0.5) |
| Lactate | 1/24 h | 4858 (95.7) | 1935 (38.1) |
| pCO2 | 1/24 h | 1626 (32.0) | 25 (0.5) |
| Blood pressure ^c^ | 1/1 h | 3480 (68.6) | 11 (0.2) |
| Heart rate ^c^ | 1/1 h | 3850 (71.9) | 3 (0.1) |
| Pulse pressure ^c^ | 1/1 h | 3480 (68.6) | 11 (0.2) |
| Respiratory rate ^c^ | 1/1 h | 4253 (83.8) | 63 (0.2) |
| Mechanical ventilation | 1/1 h | NA | 0 (0) |
| FiO2 | 1/1 h | NA | 0 (0) |
| Fever ^c^ | 1/1 h | 4867 (95.9) | 169 (4.1) |
| Urine output | 1/1 h | 4771 (94.0) | 36 (0.7) |
| Fluid balance | 1/1 h | 156 (3.1) | 6 (0.1) |
| Worsening CNS status | - | NA | NA |
| Candida respiratory culture | - | NA | NA |
| Sputum count | - | NA | NA |
| Increased insulin rate | - | NA | NA |
| Increased vasopressor rate | - | NA | NA |

Additional table 2 provides detail on the number and proportion (%) of missing data points before data preprocessing for predictors included in the final model.

Abbreviations: APACHE Acute Physiology and Chronic Health Evaluation; CNN convolutional neural network; CNS central nervous system; CRP c-reactive protein; FiO2 fraction of inspired oxygen; h hour; ICU-AI Intensive Care Unit Acquired Infection; N number; NA not applicable; pCO2 partial pressure of carbon dioxide.

^a^ The proportion of ICU admissions with ≥ 1 data point value missing. Missing values are evaluated relative to the typical sample rate for each variable before up-sampling, imputation or further preprocessing. For example, routine laboratory values are typically measured daily (i.e. 1/24 h).

^b^ The proportion of ICU admissions with no values recorded for a given variable.

^c^ Data were extracted from the ICU patient data management system at 1-minute intervals and subsequently sampled into 1-hour time bins, by taking the mean values if multiple measurements were available. Missing values are evaluated relative to the 1-hour rate for each variable.

APPENDIX D

**Additional table 3.** **Model coefficients of the Deep- LMCR model for 48-hour risk of ICU-AI**

| **Predictor** | **Beta** | **HR** | **Lower CI** | **Upper CI** | **P** |
| --- | --- | --- | --- | --- | --- |
| Landmark time | 0.79 | 2.2 | 1.6 | 3 | <0.001 |
| Landmark time squared | -0.033 | 0.97 | 0.89 | 1.1 | 0.45 |
| Male sex | 0.29 | 1.3 | 1.2 | 1.5 | <0.001 |
| Age | -0.00023 | 1.00 | 1.00 | 1.00 | 0.92 |
| Surgical admission | 0.25 | 1.3 | 1.1 | 1.5 | <0.001 |
| Readmission | -0.079 | 0.92 | 0.77 | 1.1 | 0.4 |
| Diabetes Mellitus | -0.17 | 0.84 | 0.71 | 1.00 | 0.048 |
| Immunedeficiency | 0.16 | 1.2 | 0.96 | 1.4 | 0.11 |
| Chronic corticosteroid use | 0.18 | 1.2 | 0.96 | 1.5 | 0.12 |
| Chronic organ failure | 0.07 | 1.1 | 0.93 | 1.2 | 0.34 |
| APACHE admission diagnosis category |  |  |  |  |  |
| Cardiovascular | ref | NA | NA | NA | NA |
| Gastroenterological | -0.31 | 0.73 | 0.57 | 0.94 | 0.013 |
| Neurological | 0.16 | 1.2 | 0.96 | 1.4 | 0.13 |
| Other | -0.55 | 0.58 | 0.35 | 0.95 | 0.031 |
| Respiratory | -0.34 | 0.71 | 0.59 | 0.86 | <0.001 |
| Post-transplantation | -0.021 | 0.98 | 0.75 | 1.3 | 0.88 |
| Trauma | 0.16 | 1.2 | 0.95 | 1.5 | 0.14 |
| Blood pressure | -0.0035 | 1.00 | 0.99 | 1.00 | <0.01 |
| Heart rate | 0.0038 | 1.00 | 1.00 | 1.00 | <0.01 |
| Fever | 0.58 | 1.8 | 1.6 | 2 | <0.001 |
| Urine output | -0.00099 | 1.00 | 1.00 | 1.00 | 0.11 |
| Fluid balance | -0.00011 | 1.00 | 1.00 | 1.00 | <0.01 |
| Worsening CNS status | 0.1 | 1.1 | 1.00 | 1.2 | <0.01 |
| FiO2 | 0.0092 | 1.00 | 1.00 | 1.00 | <0.001 |
| Sputum count | 0.022 | 1.00 | 1.00 | 1.00 | 0.018 |
| CRP, last value | -0.000004 | 1.00 | 1.00 | 1.00 | 0.99 |
| CRP, change | 0.005 | 1.00 | 1.00 | 1.00 | <0.001 |
| White blood cell count, last value | 0.0061 | 1.00 | 1.00 | 1.00 | <0.001 |
| White blood cell count, change | 0.0054 | 1.00 | 1.00 | 1.00 | 0.17 |
| Platelet count, last value | -0.0013 | 1.00 | 1.00 | 1.00 | <0.001 |
| Platelet count, change | 0.00011 | 1.00 | 1.00 | 1.00 | 0.86 |
| Prothrombin time, last value | 0.00063 | 1.00 | 0.99 | 1.00 | 0.93 |
| Creatinine, last value | 0.00000038 | 1.00 | 1.00 | 1.00 | 1.00 |
| Creatinine, change | -0.0016 | 1.00 | 1.00 | 1.00 | 0.031 |
| Total bilirubin, last value | 0.0017 | 1.00 | 1.00 | 1.00 | <0.01 |
| Total bilirubin, change | 0.0042 | 1.00 | 1.00 | 1.00 | 0.021 |
| Bicarbonate, change | -0.0084 | 0.99 | 0.98 | 1.00 | 0.31 |
| Lactate, last value | -0.017 | 0.98 | 0.93 | 1.00 | 0.58 |
| pCO2 change | -0.0033 | 1.00 | 0.99 | 1.00 | 0.16 |
| Candida respiratory culture | -0.18 | 0.84 | 0.74 | 0.94 | <0.01 |
| Increased insulin rate | 0.081 | 1.1 | 1.00 | 1.2 | 0.047 |
| Increased vasopressor rate | 0.31 | 1.4 | 1.3 | 1.5 | <0.001 |
| Mechanical ventilation | 0.16 | 1.2 | 1.00 | 1.4 | 0.043 |
| Pulse pressure | 0.0002 | 1.00 | 1.00 | 1.00 | 0.88 |
| Respiratory rate | 0.015 | 1.00 | 1.00 | 1.00 | <0.001 |
| Oxygen saturation | -0.033 | 0.97 | 0.95 | 0.98 | <0.001 |
| CNN score | 2.2 | 8.6 | 5.2 | 14 | <0.001 |

Additional table 3 provides model coefficients and associated confidence intervals for predictors included in the final model.

Abbreviations: APACHE Acute Physiology and Chronic Health Evaluation; CNN convolutional neural network; CNS central nervous system; CRP c-reactive protein; FiO2 fraction of inspired oxygen; ICU-AI Intensive Care Unit Acquired Infection; NA not applicable; pCO2 partial pressure of carbon dioxide.

APPENDIX E

**Additional fig. 1. Flowchart of inclusions**

**
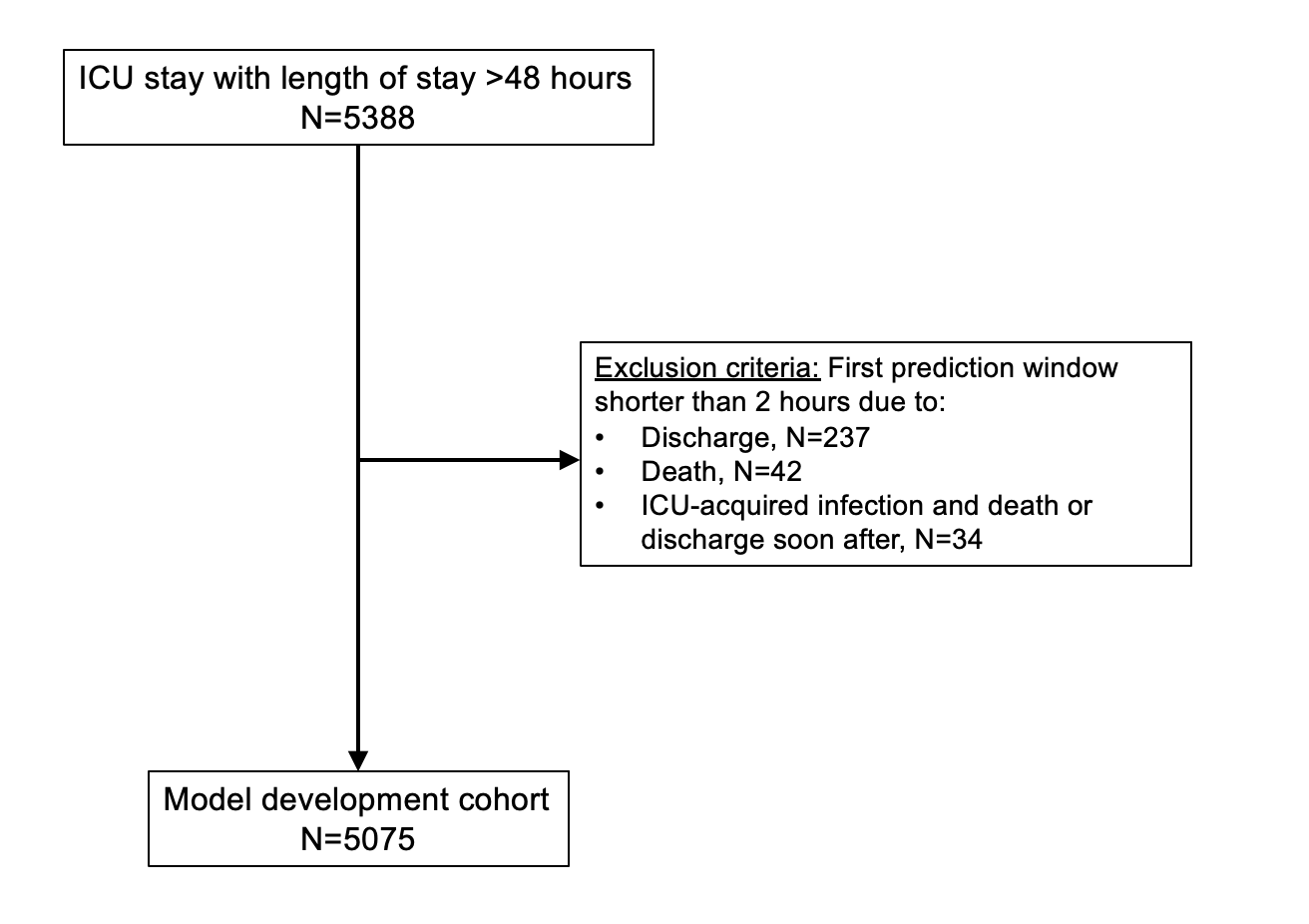
**

With the first landmark point set at t=48 hours, patients who died or were discharged in the first 2 hours of the 8-hour prediction interval (i.e. before t=48+2 hours) did not experience enough ICU time for meaningful model predictions and were excluded from analysis. Similarly, patients who developed an infection between 48-50 hours after ICU presentation and died or were discharged shortly after were excluded from the study cohort.

APPENDIX F

**Additional fig. 2 Calibration of the LMCR base model**

The grey shaded bands around the solid black line indicate the 95% confidence interval of the observed outcome proportions. To enhance interpretation, the axes were adjusted to a scale from 0.0 to 0.60, based on the low observed and estimated outcome incidences.

APPENDIX G

**Additional fig. 3. Calibration of the Deep-LMCR model**

The grey shaded bands around the solid black line indicate the 95% confidence interval of the observed outcome proportions. To enhance interpretation, the axes were adjusted to a scale from 0.0 to 0.60, based on the low observed and estimated outcome incidences.

APPENDIX H

**Additional fig. 4. Ranking of individual predictors across landmark timepoints**


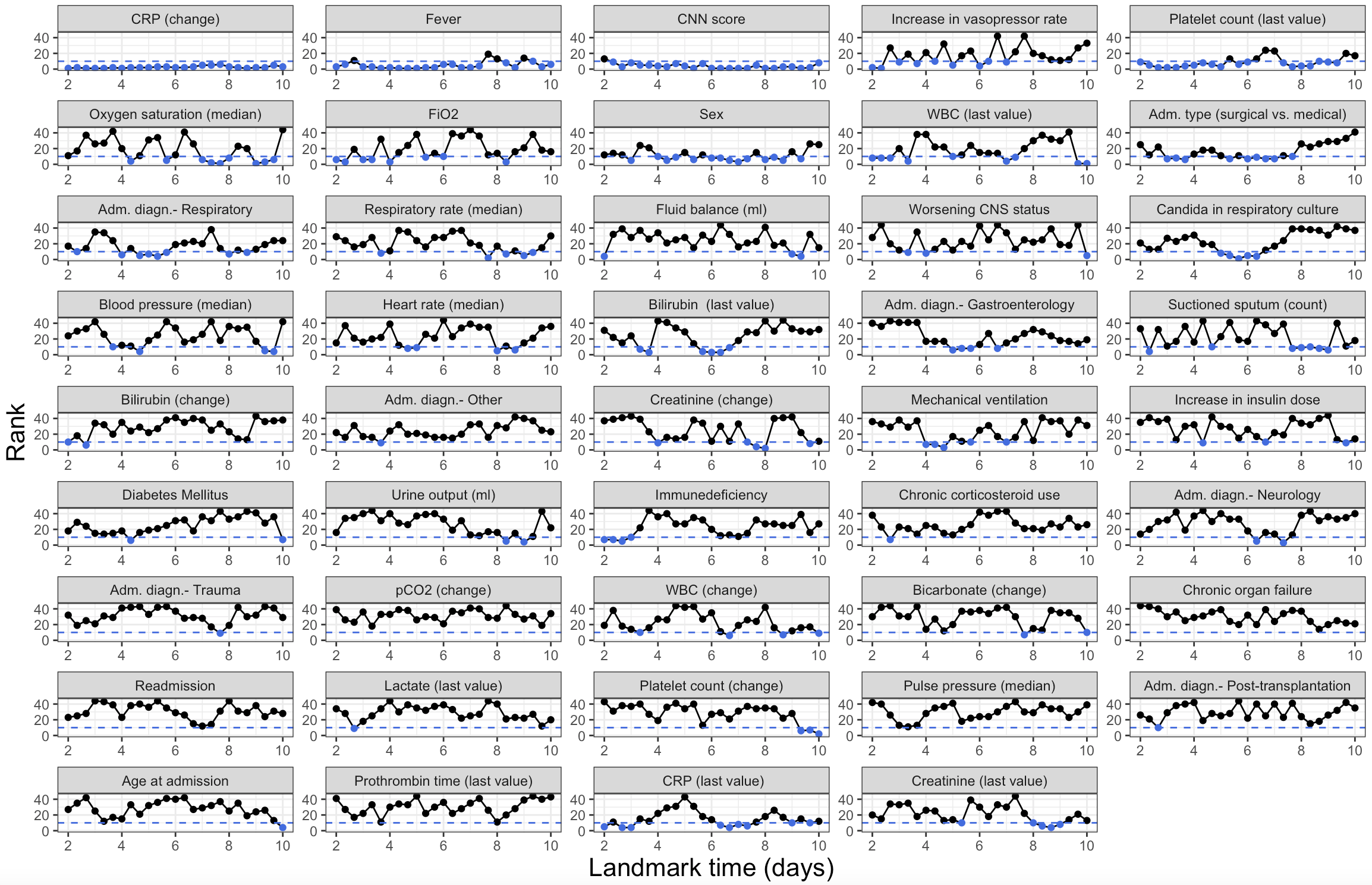


The blue points indicate timepoints when a predictor was ranked among the top 10 strongest predictors according to the Wald X2-statistic. The y axis shows the relative rank of each predictor (ranked from 1 to 40 from bottom to top). The dashed line indicates the threshold for the ten most important predictors.
