# Supplementary material for: Early detection of ICU-acquired infections using high-frequency electronic health record data
Source: BMC Med Inform Decis Mak. 2025 Jul 21;25:273. doi: 10.1186/s12911-025-03031-6 (PMC12278606; doi:10.1186/s12911-025-03031-6)
Supplement: Supplementary file 1 — Supplementary Material 1 [file 96dbfd30-ff69-4a88-93a4-4f1f84e6098a - response 1.pdf › 96dbfd30-ff69-4a88-93a4-4f1f84e6098a - response 1.pdf]

Early detection of ICU-acquired infections using high-frequency electronic health record data

RESPONSE TO REVIEWERS

We very much appreciate the Editor's and Reviewers' thorough analysis of our work and agree that the comments below offer significant opportunities for improving the presentation of our results. We have made several adjustments and additions based on the reviewers' comments (indicated in our responses below).

Reviewer Comments:

Reviewer 1
This is a prediction model with high clinical relevance and the study developed A dynamic modelling approach that incorporates machine learning of high-frequency vital sign data shows promise as a continuous bedside index of infection risk. Further validation is needed to weigh added complexity and interpretability of the deep learning model against potential benefits for clinical decision support in the ICU. I have a few comments as follows:

1. there is a lack of population selection section; how did you construct the cohort? In the present case, those with infection on admission should be excluded.

The study population consists of all patients who are admitted to the ICU for longer than 48 hours following presentation, as this is when the risk of ICU-acquired infection sets in (see line 115). However, with the first landmark point set at t=48 hours, patients who died or were discharged in the first 2 hours of the 8-hour prediction interval (i.e. before t=48+2 hours) did not experience enough ICU time for meaningful model predictions and were excluded from analysis. Similarly, patients who developed an infection between 48-50 hours after ICU presentation and died or were discharged shortly after were excluded from the study cohort.

To clarify this process for the reader, we have included a detailed patient flowchart in the Supplemental Materials (Appendix E, fig 1).

To clarify your second point: we deliberately did not exclude patients with sepsis as their reason for ICU admission, as these patients can also develop secondary infections in the ICU like any other patient. However, infectious events present at ICU admission (or those with onset before 48 hours) were not counted as outcome events in our study. To avoid misclassification, we ensured clear separation between ineligible (early) infections occurring during the first 48 hours of ICU admission and eligible (late) ICU-acquired infections by excluding all data collected during the 48-hour period following any infectious event onset.
This process is described in the Methods section, pg. 7, lines 139-155.

2. will you consider TRIPOD-AI checklist?

Unfortunately, the TRIPOD+AI checklist was only published in April 2024 after the date that we first submitted our paper to the BMC Medical Research Methodology journal. We thus did not use it for our initial submission. However, we have now checked our manuscript against the TRIPOD+AI guidelines, referenced it in the manuscript, and included the TRIPOD+AI checklist as part of the Supplemental Materials.

3. There are many factors related to the outcome being identified in the model, however their causal relationship is largely unknown; I would suggest discussion on this point in the context of causal inference since the causal association can have significant impact on clinical management.

We appreciate this expert insight and the suggestion to further contextualize our work. However, while interesting, we feel that a comprehensive discussion on causal inference in observational data is beyond the scope of this paper. We merely intended to explore a dynamic framework for early detection of ICU-AI's, which is a prediction task entirely.

4. do you have data on the site of infection? In table 1, bacteremia and CRBSI is not mutually exclusive.

The MARS cohort that was utilized for this study collected extensive data on all infections that occurred during ICU stay. As part of data registration, infection characteristics, including details about the most likely source/site of infection, were recorded for each infection event based on standardized definitions and post hoc adjudication of available clinical, radiological and microbiological information. Further details about how infections were classified are available in a paper by Klein-Klouwenberg et al. (doi: 10.1097/CCM.0b013e3182923712), which we also reference in our manuscript on page 6, line 125, reference [8].

We agree that the distinction between bacteremia and CRBSI was not clearly defined based on the information provided in table 1. We have now clarified this in a footnote reading: "*Bacteremia events were limited to primary bloodstream infections without an identifiable source during adjudication. Secondary bacteremia events, including catheter-related bloodstream infections, are listed under their respective infection sites."*.

5. In competing risk analysis, the ICU discharge can be a competing risk for the infection.

Yes, we completely agree. ICU discharge was indeed considered competing event in our competing risk model.
This is specified on line 162-165 of pages 7-8 in the revised manuscript with added detail: *"Competing outcome events for these models were death and discharge from ICU. The survival probability of ICU-AI within a 48-hour time horizon was thus estimated conditional to surviving event-free until each landmark time*."

We also refer readers interested in the mathematical details and further insight into how we constructed the competing risk landmarking model to Lancia et al. (doi: 10.1016/j.artmed.2024.102862), which is provided as reference in our revised manuscript on page 8, line 189-191, reference [12].
"Additional mathematical details about the LMCR model and the architecture of the final CNN hves been presented in more detail previously [12]."

6. The most important risk factor for ICU-AI is the long stay in ICU; with each day increase in ICU, the cumulative risk can increase. This should be accounted for during modeling.

This is a valid point, and we thank the reviewer for their knowledgeable evaluation of our work. The model accounts for prior ICU length of stay through the addition of linear and quadratic terms for landmarking time. This approach allows the predicted risk of ICU-AI onset to vary as time in ICU progresses.

We have clarified this in the revised version of the manuscript:
Page 8, lines 170-171:
"The final model included linear and quadratic terms for landmarking time to account for changing infection risk with increasing duration of ICU stay."

Reviewer 2

PEER REVIEWER COMMENTS:

GENERAL COMMENTS: The authors present a dynamic prediction model that leveraged high-frequency longitudinal data to estimate ICU acquired infection risk 48 hours ahead of clinical deterioration. Machine learning models are increasingly becoming highly relevant in predicting risk for severe disease and also in diagnosis. The study uses robust methodology to estimate dynamic risk of infections. The data have high clinical relevance and highlights the need for updating AI models with clinical data.

REQUESTED REVISIONS:
1. How was ICU AI infection defined or identified?

Please also see our reply to reviewer 1, Q4. In brief: we analyzed data that had been prospectively collected as part of the Molecular Diagnosis and Risk Stratification of Sepsis (MARS) study. As part of this project dedicated study personnel collected extensive data on all (suspected) infectious events for which systemic antibiotic therapy was started. Infection characteristics, including details about the most likely source of infection, were adjudicated according to standardized definitions. For further details on infection classification, we would like

to refer to Klein-Klouwenberg et al. (doi: 10.1097/CCM.0b013e3182923712), which is added as a reference to our manuscript on page 6, line 125, reference [8].

In our current analysis, the reference time for infection diagnosis was defined as the earlier of either the start of (empirical) antibiotic therapy or the collection of a blood culture that subsequently yielded positive results. This is described in the methods section of the manuscript, page 6, lines 122-127.

2. Line 158: Landmarking survival model estimated the risk of ICU-AI onset within the next 48 hours, was it next 48 hours after 48 hours of ICU presentation or from ICU presentation ?

Thank you for pointing out how this might need more clarification. The first landmarking timepoint at which a prediction was generated occurred at t=48 hours after ICU presentation (page.7, line 148). Consequently, the first time-window for which the risk of ICU-AI onset was estimated extended from 48 to 96 hours. Figure 1 gives the reader a visual representation of the modelling framework. To help the reader, we now specify in the legend of figure 1 more clearly that the first landmarking started 48 hours after ICU presentation (line 451-452 of the manuscript).

3. Line 161: "the patient subset at risk at that time point" does this mean at every 8 hour time period when the patient in in risk of future infection .

Yes, this is correct. Patient time series were partitioned into 8-hour intervals. To clarify this for the reader the revised text (page 7, lines 162) now reads:
"We constructed a series of independent models for each landmark which optimally leveraged the predictive information contained in the patient subset at risk at that time point (i.e., patients still residing in the ICU and at risk of future infection at each 8-hourly landmark)."

4. How was case fatality and ICU mortality rates calculated?

Case fatality was calculated as the proportion of study patients who died within 10 days of their initial ICU presentation. We specifically elected to report mortality at day 10 because most ICU-AI and competing risk events (75%) in our cohort occurred within the first 10 days, suggesting that indeed most relevant outcomes were captured within this time window. In addition, previous studies have used this cutoff to define prolonged ICU stay, which can be a turning point for ICU outcomes. Total ICU mortality was calculated as the proportion of study patients who died during their ICU stay. Of note, both estimates exclude early deaths (within 48 hours) as these patients were never at risk for ICU-acquired infection and thus ineligible for this study.

5. What was the median time for onset of recurring infections?

The median time until onset of a recurring infection was 14.5 days (IQR 12-18). We have included this information in the manuscript on page 10, lines 225-226:
"The first episode of ICU-AI occurred after a median of 7 (interquartile range (IQR) 5-9) days, whereas recurring infection occurred after a median of 14.5 (IQR 12-18) days."

6. Line 239: who were the high-risk patients ?
Thank you for requesting clarification here– we see how this might be ambiguous to the reader. This refers to patients at highest predicted risk of ICU-AI in the context of model overfitting and calibration. Overfitting tends to manifest as predicted risks that are too extreme (i.e. patients at high risk of the event tend to get overestimated risk predictions, whereas patients at low risk of the event tend to get underestimated risk predictions).

We have revised the sentence in the following way to indicate that overestimated predictions generally occurred in patients with highest predicted risk of ICU-AI rather than "high-risk patients" (pg 10, lines 242-245):
"Calibration assessment across various prediction moments indicated some overestimation of the probability of ICU-AI overall (calibration intercepts between -0.02 and -0.44), most notably in patients with highest predicted risk (calibration slopes between 0.4 and 0.66) (Additional fig 1)."

7. Predictors that showed higher strength when were they measured?

Details about individual predictors and how they were measured are provided in the Supplemental Materials, Appendix B- Additional table 1. In addition, Appendix A in the Supplemental Materials gives explanation on how data were preprocessed.
Predictors that consistently displayed high predictive strength in our model included a rise in CRP, fever, and lower platelet counts. The predictor "fever" was based on body temperature, which was defined as the presence of fever (>38 degrees Celsius) in the preceding 8 hours. A rise in CRP and decreasing platelet count were based on the change between the last two available laboratory measurements. The clinical workflow in our ICU includes daily laboratory measurement of standard chemistry, complete blood count and inflammatory marker values with additional measurements as indicated by treating physicians. A predictor, such as change in CRP or platelet count, would therefore generally be based on measurements from the preceding 24 hours.
