# Supplementary material for: Early detection of ICU-acquired infections using high-frequency electronic health record data
Source: BMC Med Inform Decis Mak. 2025 Jul 21;25:273. doi: 10.1186/s12911-025-03031-6 (PMC12278606; doi:10.1186/s12911-025-03031-6)
Supplement: Supplementary file 1 — Supplementary Material 1 [file 96dbfd30-ff69-4a88-93a4-4f1f84e6098a - response 2.pdf › 96dbfd30-ff69-4a88-93a4-4f1f84e6098a - response 2.pdf]

Reviewer Comments:

Reviewer 1
my previous comments are well addressed.
We thank the reviewer for their time and insights.

Reviewer 2
GENERAL COMMENTS:
The authors have tried to address most of my comments apart from comment 4 and 7, please see specific comments below.
Thank you for taking the time to review the revised manuscript. We hope to address the remaining concerns below and in the newly revised manuscript and supplemental materials.

REQUESTED REVISIONS:
The authors have not given a proper clarification about calculating mortality rate . While they can calculate CFR , mortality rate cannot be calculated from this type of study and hence needs to be removed.
We appreciate this insight and understand how total mortality may not be appropriate in the context of a landmarking model. We have removed total mortality from the results section of the manuscript as suggested.

Comment 7: authors states " The predictor “fever” was based on body temperature, which was defined as the presence of fever (>38 degrees Celsius) in the preceding 8 hours" preceding to what, was this a dynamic process or just measured once referring to preceding 8 hours to admission, this needs clarification?
Thank you for pointing out this out. Body temperature was measured as a dynamic repeated measurement throughout ICU stay (in most cases as a continuous measurement of core body temperature). The predictor “fever” was thus based on dynamic temperature measurements in the 8 hours *preceding landmark time*. We have clarified this in the Supplemental Materials, Appendix B-table 1, which provides details about all individual predictors.
